# Integrated Care in the Context of a Changing Environment – does it Matter?

**DOI:** 10.5334/ijic.7514

**Published:** 2022-11-30

**Authors:** K. Viktoria Stein

**Affiliations:** 1 Integrating Health and Care, Leiden University Medical Centre, Netherlands

**Keywords:** environmental impact, health and wellbeing, climate change, climate-sensitive health systems, integrated care

In an unprecedented effort last September [1] over 200 health journals, including the International Journal of Integrated Care, published an editorial with an urgent call to action to politicians and governments to implement immediate measures to control the rise in temperature, restore nature and address the health impact of climate change. Based on the numbers, the urgency is evident, e.g.:

- Every year, environmental factors take the lives of around 13 million people [2]. In comparison, non-communicable diseases kill 41 million people each year, 74% of all deaths [3].
- Over 90% of people breathe unhealthy levels of air pollution, largely resulting from burning fossil fuels driving climate change. In 2018, air pollution from fossil fuels caused USD 2.9 trillion in health and economic costs, about USD 8 billion a day [4].
- The direct damage costs to health is estimated to be between USD 2–4 billion per year by 2030 [5].
- An estimated 40% of the global population will be living in areas under severe water stress by 2050, and approximately five million more deaths will be attributable to climate change [6].
- The International Labour Organization forecasts that the equivalent productivity of 80 million full-time jobs will be lost by 2030 due to heat-related stress [7].

The environment we live in has always shaped our societies, as well as our health and wellbeing, but for a long time, the impact of environmental factors on the health and wellbeing of individuals and populations has been largely ignored. Well-known environmental risk factors, such as water or air pollution have typically been regarded under the remit of development corporation rather than global health and wellbeing. But the publication of the UN Sustainable Development Goals [8] and the ever-growing evidence on climate change has shone a light on the relationship we have with our environment, and as a consequence the effect it has on our health and wellbeing. Nonetheless, it is still rare to find climate and health experts come together to discuss how a sustainable lifestyle can benefit both; the notable exception being the Lancet Countdown on health and climate change, which published its seventh report in 2022 [9]. But has this anything to do with integrated care? Do we really need to take on another crisis, when we are in constant crisis mode already? Is it even feasible, if many of these challenges need a global response?

Higher temperatures affect our ability to be physically active, work, or learn. More extreme weather also means putting our food chains in jeopardy, which already influences what produce is being planted and how much it yields. Taken together this added stress and trauma can decrease the resilience of individuals and communities, and exacerbate pre-existing conditions; poor insulation and inadequate housing can make home-based care unsafe; soring food prices can lead to even unhealthier diets. In the face of these developments concepts such as Global Health [10], Planetary Health [6], and One Health [11] have emerged to address the interrelationship between our natural and social environments and our individual and collective health. It is interesting to note, that these concepts have their roots in the movements towards decolonisation of health and health equity, and received a considerable boost with the publication of the UN Sustainable Development Goals. They all call for inter-disciplinary, cross-sectoral and integrated action to protect our health and wellbeing and the environment we live in.

Thus, while efforts to understand and control environmental risk factors on the global level are growing, the question of how to address them locally should become a high priority topic as well. One answer to these challenges was recently given by the WHO, which described so-called climate-sensitive health risks, including for example injuries and mortality from extreme weather events, malnutrition and food-borne diseases, or mental and psychosocial ill health [5]. As always, climate change impacts the most vulnerable populations the hardest, but the detrimental effects can be felt by everyone, as social and environmental determinants of good health, such as clean air, sufficient food or secure shelter, are eroded and health systems face increasingly complex and rapidly changing crises.

So what does this all mean for integrated care? By and large, integrated care models address the burden of NCDs using a holistic understanding of health and wellbeing, and moving away from a disease-focussed to a person-centred approach. On the evolutionary path of integrated care, the more mature models and countries are already including sectors and professions outside of the traditional primary/secondary care dyad, extending their concepts to include housing, transport or education. Often cited examples, also in this journal, are the Healthy Homes and Neighbourhoods approach in Sydney [e.g. 12] or the whanau ora approach in Aotearoa New Zealand [13]. In addition, a key tenet of most integrated care models is health promotion and prevention, emphasising physical activity and healthy nutrition. By extension, these approaches are not only healthier for ourselves, but also for the environment: taking a bike is better than driving a car, and eating fresh, local, in-season produce is more sustainable than fast food. And like Global Health or One Health, integrated care calls for community participation to build healthier, more resilient neighbourhoods.

Obviously, there are clear parallels and links between integrated care and climate action, but how can we meaningfully synergise and put them into practice? On the system level, Wales has become one of the first countries to conduct a Health Impact Assessment of Climate Change, outline the measures to be taken to climate-proof the system and demonstrate how this will support health and wellbeing [14]. On the organisational level, this means e.g. looking at your local infrastructure (e.g. hospitals, public buildings, homes) and analysing together with your integrated care network, whether you are adequately equipped to provide the right care at the right time in a climate-safe space. For professionals, it is even more important than ever to understand the social determinants of health, collaborate across sectors and practice a person-centred approach to service provision, taking the possible impact of climate change into account. People and communities have already demonstrated their capacity for climate action, now it is high time to bridge the gap and connect climate action with action for healthier communities. As has been called for so often in the integrated care community, health literacy and co-designed approaches need to be at the core of these efforts. And on all levels, it is paramount to acknowledge the inextricable relationship between mental and physical health.

Within our integrated care community, calls for action have been manifold and constant, whether it is for integration with sectors outside of health, the move away from disease management towards health promotion, or for an honest effort at co-design and community participation across all levels. Change has been slow so far, and the COVID-19 pandemic has only temporarily accelerated the pace. If integrated care is a means to improve the health and wellbeing of individuals and communities taking a holistic approach, then environmental risk factors including from climate change need to be on our agenda as a contextual factor at the least. Unlike most risk factors for non-communicable diseases or most social determinants of health, climate change is irreversible, but as with the aforementioned, individual and community action can change the impact on health and wellbeing. As such, climate change is one more argument to promote integrated, person-centred, community-based care as a means to address the ever-more complex challenges of our times.
